# Assessment of In Vitro Cefiderocol Susceptibility and Comparators against an Epidemiologically Diverse Collection of *Acinetobacter baumannii* Clinical Isolates

**DOI:** 10.3390/antibiotics11020187

**Published:** 2022-01-31

**Authors:** Clara Ballesté-Delpierre, Ángel Ramírez, Laura Muñoz, Christopher Longshaw, Ignasi Roca, Jordi Vila

**Affiliations:** 1 Department of Clinical Microbiology, ISGlobal, Hospital Clínic—Universitat de Barcelona, Rosselló 149-153, 08036 Barcelona, Spain; clara.balleste@isglobal.org (C.B.-D.); aramirma47@alumnes.ub.edu (Á.R.); laura.munoz@isglobal.org (L.M.); 2 CIBER Enfermedades Infecciosas, 28029 Madrid, Spain; 3 Infectious Diseases, Shionogi B.V., London WC2B 6UF, UK; christopher.longshaw@shionogi.eu

**Keywords:** *Acinetobacter baumannii*, antibiotic resistance, carbapenem, MDR, trojan horse, cefiderocol, siderophore, epidemiology

## Abstract

Cefiderocol is a catechol-substituted siderophore cephalosporin combining rapid penetration into the periplasmic space with increased stability against β-lactamases. This study provides additional data on the in vitro antimicrobial activity of cefiderocol and commercially available comparators against an epidemiologically diverse collection of *Acinetobacter baumannii* clinical isolates. Antimicrobial susceptibility was tested using pre-prepared frozen 96-well microtiter plates containing twofold serial dilutions of: cefepime, ceftazidime/avibactam, imipenem/relebactam, ampicillin/sulbactam, meropenem, meropenem/vaborbactam, ciprofloxacin, minocycline, tigecycline, trimethoprim/sulfamethoxazole and colistin using the standard broth microdilution procedure in cation-adjusted Mueller–Hinton broth (CAMHB). For cefiderocol, iron-depleted CAMHB was used. A collection of 113 clinical strains of *A. baumannii* isolated from Argentina, Azerbaijan, Croatia, Greece, Italy, Morocco, Mozambique, Peru and Spain were included. The most active antimicrobial agents against our collection were colistin and cefiderocol, with 12.38% and 21.23% of non-susceptibility, respectively. A high proportion of multidrug-resistant (76.77%) and carbapenem-resistant (75.28%) *A. baumannii* isolates remained susceptible to cefiderocol, which was clearly superior to novel β-lactam/β-lactamase inhibitor combinations. Cefiderocol-resistance was higher among carbapenem-resistant isolates and isolates belonging to ST2, but could not be associated with any particular resistance mechanism or clonal lineage. Our data suggest that cefiderocol is a good alternative to treat infections caused by MDR *A. baumanni*, including carbapenem-resistant strains.

## 1. Introduction

The emergence of bacteria resistant to currently available antibiotics is steadily increasing but the development of new therapies to combat infections caused by these microorganisms are not following at an appropriate pace. In the clinical setting infections caused by multidrug-resistant (MDR), Gram-negative bacteria are steadily increasing and of major global concern, particularly those caused by clinical isolates of Enterobacterales and non-fermentative Gram-negative bacilli, mainly *Pseudomonas aeruginosa* and *Acinetobacter baumannii*. Among the antibiotics towards which these bacteria show a resistant phenotype, carbapenem resistance deserves special attention as carbapenems are used as a therapy of last resort [1].

In 2017, the World Health Organization published a global priority report of antibiotic-resistant microorganisms to guide research and development of new antibacterial agents. Carbapenem-resistant *A. baumannii* is on top of this list [2]. According to the 2018 EARS-Net publication, more than half of the *Acinetobacter* spp. isolates reported by EU/EEA countries were resistant to at least one of the three antimicrobial groups: fluoroquinolones, aminoglycosides and carbapenems (representing 36.2% for fluoroquinolone-resistance and 31.9% in the other two remaining groups) [3]. Data from the Centre for Disease Control (CDC) indicate that annually, 8500 infections are caused by carbapenem-resistant *Acinetobacter* in the United States [4]. Current therapeutic options for the treatment of carbapenem-resistant *A. baumannii* infections are limited and suboptimal, being restricted to available antibiotics including colistin, tigecycline, minocycline, amikacin and sulbactam. These antimicrobials are administered either alone or in combination, despite the occurrence of pharmacokinetic issues and high toxicity in some cases, as it is the case for colistin and amikacin [5].

Several strategies to discover new antibacterial agents are currently being explored, such as the chemical modification of existing antimicrobial agents [6]. Among these, cefiderocol (Shionogi Inc.) is likely one of the most promising molecules recently released [7]. Cefiderocol is a novel catechol-substituted siderophore cephalosporin that combines rapid penetration into the periplasmic space via iron transport and increased stability to enzymatic hydrolysis by all Ambler classes of β-lactamases. In vitro, preclinical and clinical studies have shown expanded activity against MDR bacteria compared to commercialized antibiotics [8]. In November 2019, cefiderocol was approved for the treatment of adults with complicated urinary tract infections by the Food and Drug Administration (FDA) [9] and, in May 2020, for the treatment of complicated Gram-negative infections by the European Medicines Agency [10].

In the current study, a collection of 113 *A. baumannii* clinical isolates including carbapenem-resistant and MDR isolates from nine countries across the globe was tested against cefiderocol and relevant comparative antibacterial agents following the Clinical and Laboratory Standards Institute (CLSI) broth microdilution method. These isolates were characterized from an epidemiological point of view as well as in terms of carbapenem-resistance and the carriage of carbapenem-resistant genes.

## 2. Results and Discussion

### 2.1. Susceptibility Profiles against Cefiderocol and Comparators

The present study comprised a collection of 113 bacterial isolates of *A. baumannii* recovered from several countries all over the world including both carbapenem-resistant and carbapenem-susceptible isolates. Upon testing the antimicrobial susceptibility to a selected panel of antibacterial agents, the lowest MIC_50_ values reported among all 113 isolates tested corresponded to cefiderocol and colistin (MIC_50_ = 0.5 µg/mL), closely followed by tigecycline and minocycline (MIC_50_ = 2 and 4 µg/mL, respectively). The MIC_90_ values, however, showed a slightly different pattern. Tigecycline showed the lowest MIC_90_ value (MIC_90_ = 4 µg/mL) followed by colistin (MIC_90_ = 8 µg/mL). Cefiderocol, on the other hand, presented a MIC_90_ of >64 µg/mL, only comparable to that of ampicillin/sulbactam (Table 1). While these values were above the range detected in previously published studies, Isler et al. have shown important differences in the reported MIC_50-_MIC_90_ values depending on the collection of strains being tested [5].

Nevertheless, the most active antimicrobial agents in our collection of isolates were both colistin and cefiderocol, showing only 12.38% of resistant isolates and 21.23% of non-susceptible isolates, respectively. Minocycline also showed good activity overall with 23.89% of resistant isolates. It is worth mentioning that more than 80% of the isolates were resistant to all of the remaining antimicrobial agents and only a handful of isolates showed an overall susceptible profile (Table 2). As expected, a very high proportion of isolates were non-susceptible to cephalosporins, regardless of the carriage of any carbapenemase or meropenem susceptibility, with 86.76% (98/113) of the isolates being non-susceptible to cefepime and 94.69% (107/113) showing MIC values of ceftazidime/avibactam ≥16 µg/mL.

As much as 87.61% (99/113) of the *A. baumannii* isolates included in this study were non-susceptible to at least one antimicrobial agent from three classes tested (13 of them being already resistant to colistin) and, hence, were considered as MDR [11]. Among MDR isolates, 76.77% (76/99) were susceptible to cefiderocol with MIC values of ≤8 µg/mL, but all isolates that were resistant to cefiderocol were also MDR. In addition, 72.72% (72/99) and 92.92% (92/99) of MDR isolates were also susceptible to minocycline and tigecycline, respectively, both of them being considered as valuable therapeutic alternatives for the treatment of infections caused by MDR *A. baumannii* [12]. Among minocycline-resistant *A. baumannii,* three isolates were resistant to cefiderocol but remained susceptible to tigecycline and colistin. The few tigecycline-resistant isolates were all susceptible to cefiderocol and only two of them showed resistance to colistin (Table 2).

### 2.2. Activity of Cefiderocol against Carbapenem-Resistant A. baumannii

Of the 113 isolates tested, 89 (78.76%) were resistant to meropenem (MIC≥8 µg/mL) and such resistance was associated with the carriage of either an OXA-type carbapenemase (79/89) or an OXA-type plus and NDM-type carbapenemase (9/89) in all isolates but one, which was negative for all carbapenemase-encoding genes tested and presented a carbapenem MIC of 8 µg/mL, right at the resistance breakpoint (Table 2). All the remaining meropenem non-resistant isolates were also negative for the presence of carbapenemase-encoding genes except for a single isolate carrying *bla*_OXA-24_ that showed a carbapenem MIC of 0.5 µg/mL, thus suggesting very low expression of this carbapenemase. In addition, all carbapenem-resistant *A. baumannii* isolates but one showed MIC values of meropenem/vaborbactam and imipenem/relebactam of ≥16 µg/mL, suggesting little to no activity of these β-lactam/β-lactamase inhibitors. Vaborbactam and relebactam are novelβ-lactamase inhibitors active against class A and class Cβ-lactamases but are not able to inhibit class B or D carbapenemases, which are common in carbapenem-resistant *A. baumannii* isolates [13]. The only carbapenem-resistant isolate showing reduced MICs ofmeropenem/vaborbactam and imipenem/relebactam (MIC of 2 µg/mL and 1 µg/mL, respectively) was negative for *bla* genes encoding class B or class D carbapenemases, thus suggesting the presence of additional resistance mechanisms such as expression of a class A carbapenemase that would be inhibited by the β-lactamase inhibitor in the combination (*bla*_PER_ or *bla*_VEB_ were not detected in this isolate either).

On the other hand, 75.28% (67/89) of carbapenem-resistant isolates were susceptible to cefiderocol, clearly showing superior activity of cefiderocol compared to the novel β-lactam/β-lactamase inhibitors. Interestingly, all cefiderocol non-susceptible isolates (24/113) were also resistant to meropenem except for two isolates, one carrying an OXA-40 oxacillinase and the other carrying a PER-1 class A β-lactamase (Table 2). Carriage of an OXA-23 oxacillinase (13/24, 54.16%) was the main mechanism associated with carbapenem-resistance among these isolates, closely followed by carriage of OXA-40 (8/24, 33.33%) and OXA-72 (2/24, 8.33%). These findings were in line with previous studies in which the highest MICs to cefiderocol (MIC ≥8 µg/mL) were related toisolates harbouring OXA-23 and/or OXA-40, although such results were likely biased by the fact that these two mechanisms are by far the most common mechanisms of resistance among carbapenem-resistant clinical isolates of *A. baumannii*, and it is important to highlight that the only carbapenem-susceptible isolate lacking an oxacillinase within this group presented cefiderocol MIC values of> 64 µg/mL [7,14,15,16]. In line with the above, the main carbapenem-resistant mechanism detected among cefiderocol-susceptibleisolates in our study was also OXA-23, present in 52.8% of the isolates (47/89), followed again by OXA-40 (9/89; 10.11%), OXA-72 (5/89; 5.51%) as well as a few isolates harbouring OXA-58 and OXA-253 (3.37% and 2.24%, respectively).

Interestingly, Kohira et al. recently suggested that NDM might have a role in cefiderocol non-susceptibility among Enterobacterales and PER might also be involved in a slight MIC increase of cefiderocol in *A. baumannii*, although the expression of PER enzymes alone did not seem to result in cefiderocol MIC values of >8 µg/mL and PER-positive *A. baumannii* isolates in such a study were closely clonally related [17]. Notably, only one out of nine isolates harbouring OXA-23/NDM in our study developed resistance to cefiderocol and only 5 out of 24 cefiderocol non-susceptible isolates were positive for *bla*_PER_, all but one associated to sequence type 2 (ST2). We also identified a sixth PER-positive meropenem-resistant *A. baumannii* isolate in our collection that, nevertheless, showed a cefiderocol MIC of 2 µg/mL (Table 2). The distribution of OXA, NDM and PER enzymes between resistant and susceptible isolates to cefiderocol in our collection, therefore, seemed to suggest that there is no link between cefiderocol-resistance and the carriage of a particular β-lactam-resistance gene.

### 2.3. Geographical and Epidemiological Distribution of Cefiderocol-Resistant Isolates

**Figure 1:**
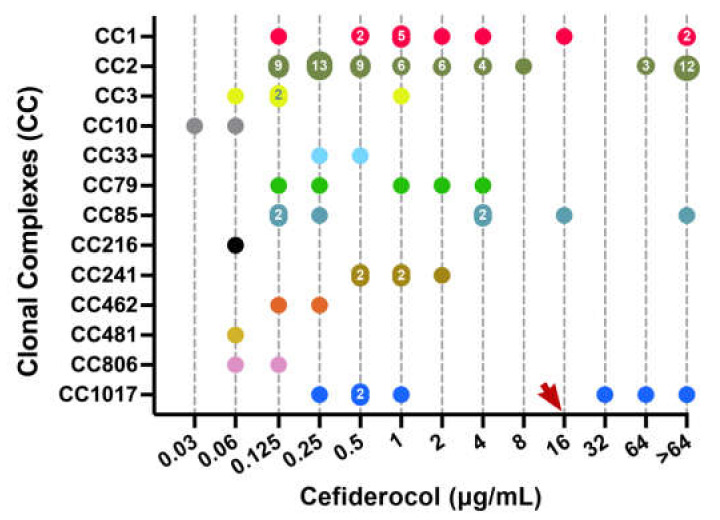
Distribution of all isolates according to their cefiderocol MIC and classification into main clonal complexes (CC). Numbers within data points indicate the number of isolates with the same MIC value. The red arrow shows the CLSI clinical breakpoint for cefiderocol (≥16 µg/mL).

*A. baumannii* isolates selected in the present study originated from nine different countries from four different continents in an attempt to provide some degree of epidemiological diversity. In addition, isolates could be grouped in at least 29 different STs from 13 clonal complexes, including isolates from the widespread international clonal complexes CC1, CC2, and CC3 (Table 2) [18]. Interestingly, however, while cefiderocol-susceptible isolates were recovered from all nine participating countries, cefiderocol non-susceptible isolates were only recovered from six out of the nine countries included, and there was a clear predominance towards isolates recovered from Azerbaijan (12/24, 50%) as well as towards isolates belonging to CC2 (15/24, 62,5%), as shown in Figure 1 and Table 2. According to PFGE data, however, there was little clonal homogeneity among the cefiderocol-resistant isolates from Azerbaijan (data not shown) and, in general, there was a strong bias in our collection towards isolates belonging to CC2 as well (62/113, 54.8%), which is just a mere reflection of the worldwide spread of such international clone [18]. For instance, we did not detect cefiderocol-resistance among isolates recovered from Peru, Argentina or Mozambique, despite some of them also belonging to CC2.

In view of the above, it is difficult to associate cefiderocol-resistance with a particular clonal lineage, although there seemed to be a certain preference in our study towards isolates from CC2. In this regard, Malik et al. also suggested an association between *A. baumannii* ST2 isolates and cefiderocol-resistance, linked to the reduced expression of the bacterial siderophore receptor PirA [19]. While the expression of siderophore receptors in different clonal groups has not yet been investigated in our group, it is likely that the emergence of cefiderocol resistance is strain specific and that the putative association with isolates from CC2 arises from the fact that mutations affecting such expression are most likely to be identified first among isolates from predominant clonal lineages, that are far more abundant in clinical settings. Nevertheless, resistance mechanisms in *A. baumannii* have typically been shown to be multifactorial, often involving modifications on membrane permeability and moiety but also biofilm formation, so additional epidemiological and molecular studies are clearly needed to elucidate the mechanisms behind cefiderocol resistance [20,21].

## 3. Materials and Methods

### 3.1. Antimicrobial Agents

Antibiotics tested in this study were previously prepared in frozen 96-well microtiter plates containing twofold serial dilutions of: cefepime, cefiderocol, ceftazidime-avibactam, imipenem-relebactam, ampicillin-sulbactam, meropenem, meropenem-vaborbactam, ciprofloxacin, minocycline, tigecycline, trimethoprim-sulfamethoxazole and colistin. The same plates and conditions were used for all strains. In all cases except for cefiderocol, the broth microdilution procedure was performed in cation-adjusted Mueller–Hinton broth (CAMHB). For cefiderocol iron-depleted, CAMHB was used and prepared following CLSI-approved methodology [22].

### 3.2. Bacterial Isolates

A collection of 113 epidemiologically diverse clinical isolates of *A. baumannii* recovered from Argentina (n = 4), Azerbaijan (n = 23), Croatia (n = 3), Greece (n = 10), Italy (n = 7), Morocco (n = 17), Mozambique (n = 14), Peru (n = 11) and Spain (n = 24) were selected for this study. For the interpretation of the results, available CLSI breakpoints were applied to determine categories (susceptible, intermediate and resistant) [22]. The *A. baumannii* ATCC 19606, *P. aeruginosa* ATCC 27,853 and *E. coli* ATCC 25,922 were used as quality control strains.

### 3.3. Minimum Inhibitory Concentration (MIC) Determinations

Antimicrobial susceptibility was performed according to the standard CLSI methodology [22]. After inoculation plates were placed in aerobic conditions and readings were taken after 16–20 h of incubation. The MIC was determined as the lowest concentration of an individual drug that resulted in no visible growth. MIC_50_ and MIC_90_ values were also calculated and indicated the MIC value of each drug able to inhibit the growth of 50% and 90% of isolates tested, respectively.

### 3.4. Molecular Identification of Carbapenem-Resistance Genes

The presence of the serine class A carbapenemases (*bla*_KPC_, *bla*_PER_ and *bla*_VEB_), class B MBLs (*bla*_NDM_, *bla*_IMP_, *bla*_VIM_, *bla*_SPM_, and *bla*_SIM_) and class D oxacillinases (*bla*_OXA-51_-like, *bla*_OXA-23_-like, *bla*_OXA-24_-like, *bla*_OXA-58_-like, *bla*_OXA-143_-like, and *bla*_OXA-235_-like) was checked by PCR, as previously described [23,24].

### 3.5. Multi-Locus Sequence Typing (MLST)

MLST was performed using the Pasteur scheme for *A. baumannii* [25]. The allele sequences and STs of selected strains were identified and retrieved from the PubMLST *A. baumannii* MLST database (http://pubmlst.org/abaumannii/ (last accessed on 17 December 2021). The population structure of STs was assigned to their corresponding clonal complexes (CCs) using the goeBURST software (http://www.phyloviz.net/goeburst/ (last accessed on 17 December 2021)).

## 4. Conclusions

Overall, almost 80% of *A. baumannii* isolates in our study showed susceptibility to cefiderocol, and, more interestingly, as many as 75% of meropenem-resistant isolates were also susceptible to the new drug. Despite the fact that most cefiderocol-resistant isolates belong to CC2, our data seem to suggest that cefiderocol-resistance is strain specific and cannot be related to any particular mechanism of carbapenem-resistance either.

There are already several publications that have investigated the in vitro activity of cefiderocol against clinical isolates of *A. baumannii* [7,26,27]. Such publications, however, usually fail to consider the clonal relatedness of isolates selected for the study and, given the strong clonal structure of *A. baumannii*, is it likely that they lack in clonal diversity. In our study we have tried not to focus on including a sheer number of isolates but to provide as much clonal diversity as possible. The susceptibility profiles from our collection of epidemiologically diverse isolates harboring different mechanisms of carbapenem-resistance, nevertheless, reinforce previous results that prompted cefiderocol as a valid alternative to treat infections caused by MDR *A. baumannii*.

## Figures and Tables

**Table 1:** Minimum inhibitory concentration (MIC, µg/mL) for cefiderocol and comparators against a collection of 113 *A. baumannii* clinical isolates. Clinical and Laboratory Standards Institute (CLSI) breakpoints were applied to determine categories of susceptible, intermediate and resistant.

Antimicrobial Agents	MIC_50_	MIC_90_	Range	S	I	R
	µg/mL			n (%)		
Cefiderocol	0.5	>64	≤ 0.03 to >64	90 (79.60)	1 (0.88)	23 (20.35)
Cefepime	>16	>16	≤ 0.125 to >16	15 (13.27)	4 (3.54)	94 (83.18)
Ceftazidime/avibactam ^a^	>16	>16	≤ 0.125 to >16	NA	NA	NA
Meropenem	>16	>16	0.125 to >16	22 (19.47)	2 (1.77)	89 (78.76)
Meropenem/vaborbactam ^a^	>16	>16	0.125 to >16	NA	NA	NA
Imipenem/relebactam ^a^	>16	>16	0.125 to >16	NA	NA	NA
Ampicillin-sulbactam	32	64	≤ 2 to >64	24 (21.24)	20 (17.70)	69 (61.06)
Ciprofloxacin	>8	>8	≤ 0.125 to >8	12 (10.62)	0 (0.00)	101 (89.38)
Minocycline	4	>8	≤ 0.25 to >8	65 (57.52)	21 (18.58)	27 (23.89)
Tigecycline ^a^	2	4	≤ 0.125 to >4	NA	NA	NA
Trimethoprim-sulfamethoxazole	> 8	> 8	≤ 0.25 to >8	19 (16.81)	NA	94 (83.18)
Colistin ^b^	0.5	8	≤ 0.25 to >8	NA	99 (87.61)	14 (12.39)

^a^ No breakpoints available at CLSI; ^b^ The CLSI update released in April 2021 removed the susceptible breakpoint for colistin. Only intermediate (≤2 µg/mL) and resistant (≥4 µg/mL) breakpoints for colistin are now available; MIC_50_ indicates the MIC value at which 50% of isolates tested are inhibited; MIC_90_ indicates the MIC value at which 90% of isolates tested are inhibited. S, susceptible; I, intermediate; R, resistant. NA, not applicable.

**Table 2:** Antibiotic susceptibility and molecular characterization of *A. baumannii* bacterial isolates used in this study.

ID	Country	CFDC	CST	MIN	CIP	SXT	TGC	SAM	FEP	CZA	MEM	MEV	IPR	aCarb	MDR	ST	CC
		µg/mL															
SHG-44	Argentina	0.125	2	1	>8	>8	1	32	>16	>16	>16	>16	>16	OXA-23	MDR	1	CC1
SHG-43	Argentina	0.25	1	8	>8	>8	2	32	>16	>16	>16	>16	>16	OXA-23	MDR	2	CC2
SHG-45	Argentina	0.5	0.5	8	>8	>8	2	64	>16	16	>16	>16	>16	OXA-23	MDR	2	CC2
SHG-46	Argentina	0.125	0.25	0.25	0.125	0.25	0.125	2	2	8	0.5	0.5	0.5	ND	--	404	CC3
SHG-94	Azerbaijan	1	0.5	1	>8	>8	0.5	64	>16	16	8	2	1	ND	MDR	19	CC1
SHG-112	Azerbaijan	0.25	0.25	0.5	>8	>8	0.5	64	>16	>16	>16	>16	>16	OXA-40	MDR	78	CC1017
SHG-108	Azerbaijan	0.5	0.25	0.25	>8	0.25	0.25	64	>16	>16	>16	>16	>16	OXA-40	MDR	78	CC1017
SHG-110	Azerbaijan	0.5	0.25	0.25	>8	0.25	0.125	8	4	16	>16	>16	>16	OXA-40	MDR	1077	CC1017
SHG-109	Azerbaijan	1	0.5	4	>8	>8	1	8	>16	>16	>16	>16	>16	OXA-40	MDR	1077	CC1017
SHG-97	Azerbaijan	0.25	0.25	8	>8	>8	2	32	>16	>16	1	0.5	0.5	ND	MDR	2	CC2
SHG-104	Azerbaijan	0.06	0.5	0.25	0.25	0.25	0.125	2	2	16	0.25	0.25	0.25	ND	--	1422	CC3
SHG-105	Azerbaijan	1	4	0.25	>8	>8	0.5	8	>16	>16	0.5	0.5	0.5	ND	MDR	1422	CC3
SHG-106	Azerbaijan	0.25	0.5	4	>8	>8	1	16	>16	>16	>16	>16	>16	OXA-23	MDR	625	CC462
SHG-100	Azerbaijan	0.06	0.5	0.25	0.25	0.25	0.125	2	2	16	0.25	0.25	0.25	ND	--	578	CC806
SHG-99	Azerbaijan	0.125	1	0.25	0.125	0.25	0.25	2	4	8	0.5	0.5	0.25	ND	--	578	CC806
SHG-47	Croatia	0.25	2	8	>8	1	2	16	>16	>16	>16	>16	>16	OXA-72	MDR	2	CC2
SHG-58	Greece	0.5	0.5	1	>8	>8	2	32	>16	>16	>16	>16	>16	OXA-23	MDR	1	CC1
SHG-55	Greece	2	2	2	>8	>8	2	64	>16	16	>16	>16	>16	OXA-23	MDR	1	CC1
SHG-57	Greece	0.125	>8	>8	>8	>8	>4	>64	>16	>16	>16	>16	>16	OXA-23	MDR	2	CC2
SHG-53	Greece	0.25	0.25	>8	>8	2	2	64	>16	>16	>16	>16	>16	OXA-23	MDR	2	CC2
SHG-56	Greece	0.5	>8	>8	>8	>8	4	64	>16	>16	>16	>16	>16	OXA-23	MDR	2	CC2
SHG-54	Greece	1	>8	4	>8	>8	>4	32	>16	>16	>16	>16	>16	OXA-23	MDR	2	CC2
SHG-52	Greece	4	8	8	>8	>8	2	64	>16	>16	>16	>16	>16	OXA-23	MDR	2	CC2
SHG-65	Italy	0.5	>8	>8	>8	>8	2	64	>16	>16	>16	>16	>16	OXA-23	MDR	2	CC2
SHG-66	Italy	1	0.5	>8	>8	>8	1	64	>16	>16	>16	>16	>16	OXA-23	MDR	2	CC2
SHG-61	Italy	2	8	4	>8	>8	2	16	>16	>16	>16	>16	>16	OXA-23	MDR	2	CC2
SHG-63	Italy	2	8	4	>8	>8	2	16	>16	>16	>16	>16	>16	OXA-23	MDR	2	CC2
SHG-62	Italy	4	0.5	>8	>8	>8	2	64	>16	>16	>16	>16	>16	OXA-23	MDR	2	CC2
SHG-60	Italy	4	>8	4	>8	>8	4	16	>16	>16	>16	>16	>16	OXA-23	MDR	2	CC2
SHG-11	Morocco	1	0.25	>8	>8	>8	1	64	>16	>16	>16	>16	>16	OXA-23/NDM	MDR	315	CC1
SHG-1	Morocco	1	0.5	>8	>8	>8	2	64	>16	>16	>16	>16	>16	OXA-23	MDR	315	CC1
SHG-10	Morocco	4	0.25	4	>8	>8	0.5	64	>16	>16	>16	>16	>16	OXA-23/NDM	MDR	315	CC1
SHG-12	Morocco	0.125	1	>8	>8	>8	2	64	>16	>16	>16	>16	>16	OXA-23/NDM	MDR	2	CC2
SHG-16	Morocco	0.125	1	>8	>8	>8	2	64	>16	>16	>16	>16	>16	OXA-23/NDM	MDR	2	CC2
SHG-5	Morocco	0.25	1	>8	>8	2	0.125	32	>16	>16	>16	>16	>16	OXA-23	MDR	2	CC2
SHG-8	Morocco	0.25	2	>8	>8	>8	2	64	>16	>16	>16	>16	>16	OXA-23/NDM	MDR	2	CC2
SHG-4	Morocco	0.5	0.5	>8	>8	>8	2	64	>16	>16	>16	>16	>16	OXA-23	MDR	2	CC2
SHG-7	Morocco	0.5	0.5	>8	>8	>8	2	64	>16	>16	>16	>16	>16	OXA-23	MDR	2	CC2
SHG-13	Morocco	0.5	0.5	8	>8	>8	2	64	>16	>16	>16	>16	>16	OXA-23/NDM	MDR	632	CC2
SHG-14	Morocco	1	0.25	>8	>8	>8	2	64	>16	>16	>16	>16	>16	OXA-23/NDM	MDR	2	CC2
SHG-17	Morocco	0.5	2	>8	>8	>8	2	64	>16	>16	>16	>16	>16	OXA-23/NDM	MDR	164	CC241
SHG-9	Morocco	2	0.5	0.25	>8	0.25	0.125	16	>16	>16	>16	>16	>16	OXA-23	MDR	164	CC241
SHG-2	Morocco	4	0.5	0.25	>8	>8	0.5	16	>16	>16	>16	>16	16	OXA-23	MDR	85	CC85
SHG-6	Morocco	4	0.5	0.25	>8	>8	1	16	>16	>16	>16	>16	>16	OXA-23	MDR	85	CC85
SHG-25	Mozambique	0.03	0.5	1	>8	>8	0.5	2	8	16	0.5	0.25	0.25	ND	MDR	23	CC10
SHG-22	Mozambique	0.06	0.25	0.25	0.5	>8	0.5	4	4	>16	1	0.5	0.5	ND	--	1435	CC10
SHG-32	Mozambique	0.25	0.5	>8	>8	>8	2	32	16	>16	4	4	1	ND	MDR	2	CC2
SHG-18	Mozambique	0.5	1	>8	>8	>8	2	32	>16	>16	4	1	0.5	ND	MDR	2	CC2
SHG-29	Mozambique	1	1	2	>8	>8	2	16	2	>16	0.5	1	0.25	ND	--	2	CC2
SHG-24	Mozambique	0.06	0.5	0.25	0.125	0.25	0.125	2	1	2	0.125	0.125	0.25	ND	--	New ^a^	CC216
SHG-27	Mozambique	0.5	0.25	0.5	>8	>8	1	8	>16	>16	1	1	0.5	ND	MDR	164	CC241
SHG-21	Mozambique	1	0.25	1	>8	>8	0.5	2	>16	>16	1	0.5	0.5	ND	MDR	164	CC241
SHG-30	Mozambique	1	0.5	0.25	>8	0.25	0.5	8	>16	>16	0.5	0.5	0.25	ND	--	164	CC241
SHG-20	Mozambique	0.25	0.5	0.25	0.125	0.25	0.125	2	0.5	4	0.125	0.125	0.25	ND	--	424	CC33
SHG-19	Mozambique	0.125	0.5	0.25	>8	>8	0.25	8	>16	>16	2	2	0.5	ND	MDR	New ^a^	CC462
SHG-31	Mozambique	0.06	1	0.25	0.125	0.25	0.125	2	2	16	0.25	0.5	0.25	ND	--	New ^a^	CC481
SHG-28	Mozambique	0.125	0.25	0.25	0.125	>8	0.125	2	2	16	0.5	0.25	0.25	ND	--	1359	CC85
SHG-26	Mozambique	0.25	0.25	0.25	0.5	>8	0.125	2	4	16	0.5	4	0.25	ND	--	1359	CC85
SHG-34	Peru	0.5	0.25	0.5	>8	>8	1	16	>16	>16	>16	>16	>16	OXA-253	MDR	7	CC1
SHG-33	Peru	1	1	1	>8	>8	2	64	>16	>16	>16	>16	>16	OXA-23	MDR	1	CC1
SHG-37	Peru	0.125	0.5	8	>8	>8	2	32	>16	>16	>16	>16	>16	OXA-72	MDR	2	CC2
SHG-36	Peru	0.5	0.5	4	>8	>8	1	32	>16	>16	>16	>16	>16	OXA-72	MDR	2	CC2
SHG-40	Peru	2	0.25	8	>8	>8	2	16	16	>16	>16	>16	>16	OXA-72	MDR	108	CC2
SHG-39	Peru	0.125	0.5	0.5	>8	2	0.25	16	>16	>16	>16	>16	>16	OXA-23	MDR	3	CC3
SHG-43	Peru	0.5	2	≤0.25	0.25	22640.25	≤0.125	≤2	4	16	0.5	0.25	0.25	ND	--	273	CC33
SHG-35	Peru	0.125	0.5	0.5	>8	>8	2	2	8	16	1	1	0.25	ND	MDR	79	CC79
SHG-38	Peru	0.25	0.5	8	>8	>8	2	32	>16	>16	>16	>16	>16	OXA-72	MDR	79	CC79
SHG-41	Peru	1	0.5	1	>8	>8	2	16	>16	>16	>16	>16	>16	OXA-253	MDR	79	CC79
SHG-42	Peru	2	0.25	0.5	>8	>8	1	64	>16	>16	>16	>16	>16	OXA-23	MDR	79	CC79
SHG-82	Spain	1	0.25	0.5	>8	>8	4	16	>16	>16	>16	>16	>16	OXA-40	MDR	1	CC1
SHG-75	Spain	0.125	2	>8	>8	>8	2	64	>16	>16	>16	>16	>16	OXA-58	MDR	2	CC2
SHG-70	Spain	0.125	0.25	8	>8	>8	0.5	32	>16	16	>16	>16	>16	OXA-23	MDR	2	CC2
SHG-77	Spain	0.125	1	8	>8	>8	1	32	16	16	>16	>16	>16	OXA-58	MDR	2	CC2
SHG-88	Spain	0.125	0.5	8	>8	>8	1	16	>16	16	>16	16	>16	OXA-23	MDR	2	CC2
SHG-89	Spain	0.125	1	8	>8	>8	2	64	>16	8	>16	>16	>16	OXA-23	MDR	2	CC2
SHG-83	Spain	0.25	1	>8	>8	8	2	32	>16	>16	>16	>16	>16	OXA-23	MDR	2	CC2
SHG-78	Spain	0.25	0.5	>8	>8	>8	>4	32	>16	>16	>16	>16	>16	OXA-23	MDR	2	CC2
SHG-90	Spain	0.25	0.5	8	>8	>8	2	16	>16	>16	>16	>16	>16	OXA-23	MDR	2	CC2
SHG-72	Spain	0.25	0.25	4	>8	8	2	32	>16	>16	>16	>16	>16	OXA-40	MDR	2	CC2
SHG-74	Spain	0.25	8	2	>8	>8	4	64	>16	>16	>16	>16	>16	OXA-23	MDR	2	CC2
SHG-76	Spain	0.25	0.5	4	>8	>8	1	16	>16	16	>16	>16	>16	OXA-40	MDR	2	CC2
SHG-80	Spain	0.5	0.5	>8	>8	>8	>4	32	>16	>16	>16	>16	>16	OXA-23	MDR	2	CC2
SHG-67	Spain	1	2	>8	>8	>8	>4	16	>16	>16	8	16	>16	OXA-58	MDR	2	CC2
SHG-79	Spain	1	0.5	8	>8	>8	4	32	>16	>16	>16	>16	>16	OXA-23	MDR	2	CC2
SHG-73	Spain	2	>8	4	>8	>8	4	64	>16	>16	>16	>16	>16	OXA-23	MDR	2	CC2
SHG-68	Spain	2	2	4	>8	>8	>4	32	>16	>16	>16	>16	>16	OXA-23	MDR	2	CC2
SHG-85	Spain	2	0.25	>8	>8	>8	1	32	>16	>16	>16	>16	>16	OXA-23 *	MDR	724	CC2
SHG-69	Spain	4	0.5	4	>8	>8	>4	64	>16	>16	>16	>16	>16	OXA-40	MDR	537	CC79
SHG-81	Spain	0.125	0.5	0.5	1	0.25	0.5	16	16	>16	>16	>16	>16	OXA-40	--	374	CC85
SHG-48	Croatia	8	0.5	8	8	2	2	32	>16	0.125	>16	>16	>16	OXA-72	MDR	2	CC2
SHG-91	Azerbaijan	>64	0.25	0.25	>8	>8	0.25	64	>16	>16	>16	>16	>16	OXA-23	MDR	19	CC1
SHG-92	Azerbaijan	>64	0.25	0.25	>8	>8	0.25	32	>16	>16	>16	>16	>16	OXA-23	MDR	1423	CC1
SHG-107	Azerbaijan	32	0.5	0.25	>8	>8	0.25	>64	>16	>16	>16	>16	>16	OXA-40	MDR	78	CC1017
SHG-111	Azerbaijan	64	2	1	>8	>8	1	8	>16	16	>16	>16	>16	OXA-40	MDR	1077	CC1017
SHG-113	Azerbaijan	>64	0.25	4	>8	>8	0.5	8	>16	>16	>16	>16	>16	OXA-40	MDR	1077	CC1017
SHG-98	Azerbaijan	64	0.5	2	>8	>8	1	64	>16	>16	0.5	0.5	0.5	OXA-40	MDR	2	CC2
SHG-96	Azerbaijan	>64	0.5	8	>8	>8	2	>64	>16	>16	>16	>16	>16	OXA-40	MDR	2	CC2
SHG-103	Azerbaijan	>64	1	8	>8	>8	2	>64	>16	>16	>16	>16	>16	OXA-23	MDR	2	CC2
SHG-93	Azerbaijan	>64	8	8	>8	2	2	8	>16	>16	>16	>16	>16	OXA-40	MDR	2	CC2
SHG-102	Azerbaijan	>64	8.00	0.5	>8	>8	0.5	32	>16	>16	>16	>16	>16	OXA-40	MDR	2	CC2
SHG-95	Azerbaijan	>64	0.5	1	>8	>8	1	>64	>16	>16	>16	16	>16	OXA-23 *	MDR	2	CC2
SHG-101	Azerbaijan	>64	0.25	0.25	>8	8	0.5	8	>16	>16	0.5	1	0.5	ND *	MDR	2	CC2
SHG-49	Croatia	64	0.5	8	8	2	1	64	>16	>16	>16	>16	>16	OXA-72	MDR	2	CC2
SHG-59	Greece	16	8	2	>8	>8	4	64	>16	>16	>16	>16	>16	OXA-23	MDR	1	CC1
SHG-50	Greece	>64	0.25	>8	>8	>8	1	64	>16	>16	>16	>16	>16	OXA-23	MDR	2	CC2
SHG-51	Greece	>64	2	>8	>8	>8	2	>64	>16	>16	>16	>16	>16	OXA-23	MDR	2	CC2
SHG-64	Italy	>64	1	8	>8	>8	1	16	>16	16	>16	>16	>16	OXA-23	MDR	2	CC2
SHG-15	Morocco	64	0.5	4	>8	>8	4	>64	>16	>16	>16	>16	>16	OXA-23/NDM	MDR	2	CC2
SHG-3	Morocco	16	0.5	0.25	>8	>8	0.25	64	>16	>16	>16	>16	>16	OXA-23	MDR	85	CC85
SHG-87	Spain	>64	0.5	>8	>8	>8	2	32	>16	>16	>16	>16	>16	OXA-23	MDR	2	CC2
SHG-86	Spain	>64	0.5	1	>8	>8	2	32	>16	>16	>16	>16	>16	OXA-23 *	MDR	2	CC2
SHG-84	Spain	>64	0.25	1	>8	>8	4	64	>16	>16	>16	>16	>16	OXA-23 *	MDR	2	CC2
SHG-71	Spain	>64	0.5	1	>8	>8	4	32	>16	>16	>16	>16	>16	OXA-40 *	MDR	32	CC85
ATCC19606	USA	≤0.06	1	≤0.25	≤0.125	>8	≤0.125	≤2	2	8	0.5	0.5	0.25	ND	--	52	--
ATCC27853 ^b^	USA	0.5	1	>8	0.25	>8	>4	>64	1	2	1	0.5	0.5	ND	--	NA	--
ATCC25922 ^c^	USA	0.25	0.25	1	≤0.125	0.25	≤0.125	≤2	≤0.125	≤0.125	≤0.06	≤0.06	0.25	ND	--	NA	--

ID, strain identification number; Country, country of origin; CFDC, cefiderocol; CST, colistin; MIN, minocycline; CIP, ciprofloxacin; SXT, trimethoprim/sulfamethoxazole; TGC, tigecycline; SAM, ampicillin/sulbactam; FEP, cefepime; CZA, ceftazidime/avibactam; MEM, meropenem; MEV, meropenem/vaborbactam; IPR, imipenem/relebactam; aCarb, acquired carbapenemase; MDR, multidrug-resistant; ST, sequence type; CC, clonal complex. ND, not detected. NA, not applicable. ^a^ These isolates presented novel allelic combinations and will be assigned a novel ST number. ^b^ *Pseudomonas aeruginosa*. ^c^ *Escherichia coli*. * Positive for PCR screening of *bla*_PER_.

## Data Availability

The datasets generated by this study are available in Table 2.
